# Comprehensive risk assessment and control measures in the food service chain of hospitals nutrition department: a case study in Al-Ahsa Governorate, Kingdom of Saudi Arabia

**DOI:** 10.3389/fmicb.2025.1551446

**Published:** 2025-05-21

**Authors:** Farag Ali Saleh, Hisham Abdelmonem Mohamed, Muhammad Munir, Mutlag Mohammad Al Otaibi, Sahar Mostafa Kamel, Omar Mohammed Alsaif, Abdulaziz Ali Alqahtani, Malak Abdullah AlDabal

**Affiliations:** ^1^Food and Nutrition Sciences Department, College of Agriculture and Food Sciences, King Faisal University, Al-Ahsa, Saudi Arabia; ^2^Date Palm Research Center of Excellence, King Faisal University, Al-Ahsa, Saudi Arabia; ^3^Saudi Food and Drug Authority, Dammam Food Laboratory, Riyadh, Saudi Arabia; ^4^King Abdulaziz Hospital, Ministry of National Guard Health Affairs, Al-Ahsa, Saudi Arabia; ^5^King Abdullah International Medical Research Center, Eastern Region, Saudi Arabia

**Keywords:** hospital nutrition, food service chain, food safety, quality assurance, healthcare facilities, hygiene practices, critical control points, hazard analysis

## Abstract

**Introduction:**

Ensuring hospital food safety is essential for patient health, infection control, and public trust. This study evaluates food exposure risks in two hospitals in Al-Ahsa Governorate, Saudi Arabia, focusing on critical control points during food processing and delivery.

**Methods:**

Microbial analysis was conducted on food samples from various stages of food preparation, including raw meat, chicken, fish, and prepared meals. Temperature monitoring and chemical hazard assessments were also carried out, including pesticide residue and heavy metal analysis.

**Results:**

The microbial analysis detected specific pathogens, including *Salmonella*, *Listeria monocytogenes*, *Escherichia coli*, and *Staphylococcus aureus*, along with yeasts and molds. Total bacterial counts (TBC) in raw meat, chicken, and fish ranged from 2.5 to 5.0 log cfu/g, while prepared meals had TBCs between 1.0 and 3.0 log cfu/g. No *Salmonella* or *Listeria monocytogenes* were detected. Chemical hazards, including mycotoxins in white flour and heavy metals, were within permissible limits. However, 12 pesticides were detected, with six exceeding European Food Safety Organization limits.

**Discussion:**

Temperature monitoring revealed that hot foods cooled to unsafe levels during transport, and cold samples were not consistently maintained at SFDA-recommended temperatures. Hospitals generally comply with health regulations but improvements are necessary in temperature control and preventing chemical contamination of raw materials.

## Graphical abstract

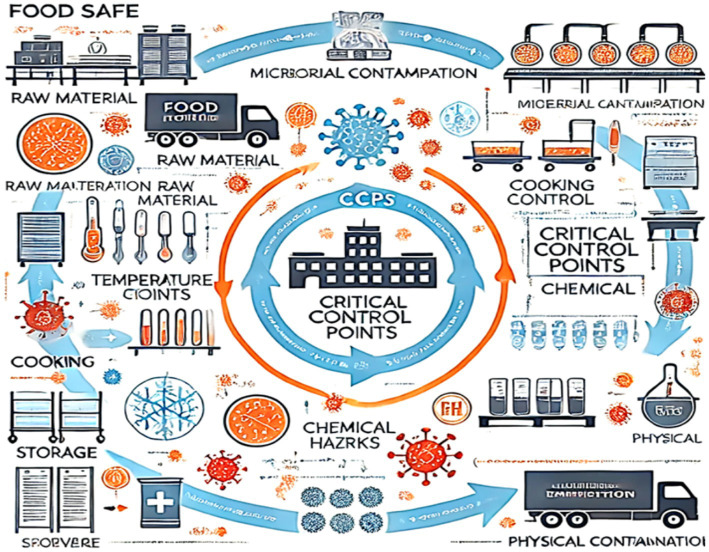

## 1 Introduction

The nutrition services department in hospitals plays a critical role in providing safe and nutritious meals to patients, involving processes such as purchasing, inspecting, receiving, and storing raw materials, as well as meal preparation and serving (Escott-Stump, 1990; Pawar and Purwar, 2013). Foodborne pathogens are a significant concern for food safety, and experts emphasize the need to control these pathogens at every stage of the food chain (Bosch et al., 2018; He et al., 2020). Contamination often arises due to worker negligence, leading to pathogenic germs, viruses, parasites, and chemicals that cause over 200 diseases, including diarrhea, food poisoning, and cancer. With approximately 600 million cases and 420,000 deaths annually, foodborne diseases pose a significant global health risk, especially to vulnerable populations such as infants, the elderly, and those with compromised immune systems (World Health Organization, 2020; Ling et al., 2021; Food and Drug Administration, 2012; Hann et al., 2023).

Contamination can spread through various sources, including food, water, utensils, and food handling practices, potentially leading to infectious and non-infectious diseases. Due to the large volume of food provided by various suppliers, hospital food is particularly vulnerable to contamination, posing risks to patients with weakened immune systems. Common bacterial pathogens like *Salmonella*, *Escherichia coli*, *Listeria monocytogenes*, and *Campylobacter* can cause gastrointestinal illnesses. Other risks include parasitic infections, molds producing aflatoxins, and chemical contaminants such as pesticides and heavy metals (Zeb et al., 2020; Bumyut et al., 2021; Suryani et al., 2021; Chea et al., 2022). Physical hazards like hair, plastic, or bones and improper management of allergens can pose serious health threats (Simothy et al., 2018; Donkor, 2019, 2020; Bushra et al., 2022). Moreover, personal hygiene, worker health status, and food safety behaviors significantly contribute to contamination risks (Bilge and Demir, 2019).

Expanding food services, especially in developing countries like the Gulf Cooperation Council (GCC), has increased foodborne diseases, underscoring the importance of stringent food safety practices (Taha et al., 2021). Food safety authorities and organizations worldwide work together to control contamination by biological, chemical, and physical agents (Yeak et al., 2022). Risk assessment methods (i.e., identifying hazards, characterizing risks, assessing exposure, and evaluating risks), help establish control measures at critical points in the food service chain (Mahoney, 2020). Critical control points (CCPs) are identified at specific stages where control is necessary to prevent contamination and ensure food safety through proper temperature control, cleaning, and supplier selection. The Hazard Analysis and Critical Control Points (HACCP) system is a science-based, preventive approach used to manage these risks, ensuring that food safety protocols are strictly followed (Richards et al., 1993; Di Renzo et al., 2015; Hulebak and Schlosser, 2002).

In Saudi Arabia, the health sector has developed significantly, with 497 hospitals nationwide, including 17 in Al-Ahsa Governorate. Despite advances, foodborne disease incidents, particularly bacterial food poisoning, remain a concern, with reports of antibiotic resistance among foodborne pathogens (Al-Mazrou, 2004; Hassan et al., 2011). The Saudi Food and Drug Authority (SFDA) oversees food safety standards and the traceability of food products, but challenges persist in ensuring the safety of hospital food (El Sheikha, 2010, 2015a, 2015b).

This study aims to evaluate food safety practices in hospitals’ nutrition services by identifying and controlling risks using the HACCP system. The specific objectives are:

- To explore the relationship between food handler practices throughout the food service chain and the likelihood of foodborne risks.
- To evaluate the efficacy of food service system procedures implemented by the nutrition department in hospitals to mitigate potential risks.
- To assess the current status of food safety provided to hospital patients.
- To improve food safety protocols in hospital nutrition departments to prevent potential hazards.

## 2 Materials and methods

### 2.1 Collection of samples

The collection of samples took 4 months, as food samples and swab samples were collected from the two hospitals of Al-Ahsa Governorate, Kingdom of Saudi Arabia (KSA) during the year 2022 (co-ordinates: 25°23′N and 49°36′E). Sixty food samples (raw and cooked) were collected from each hospital. Five hundred and twenty swab samples were collected from workers’ hands, surfaces, and appliances that come into direct contact. All food and swab samples were collected using sterilized tools at all stages of food preparation. These samples were put in Stock maker bags (Seward Stomacher Ltd., United Kingdom) and placed in 17 liters ice box freezer (Engel Coolers, Florida, United States), which were then transferred to the microbial analysis laboratory, College of Agriculture and Food Sciences, King Faisal University, Al-Ahsa, KSA.

### 2.2 Identification of Critical Control Points in the hospitals

Identifying Critical Control Points (CCPs) in hospitals generally involves a collaborative approach. In this study, CCPs were determined by a multidisciplinary team of healthcare professionals, including doctors, nurses, infection control specialists, and quality assurance personnel. A decision tree approach, as recommended by the Codex Alimentarius guidelines, was employed to assess the risks and determine which steps required control measures to prevent foodborne hazards. This methodology aligns with the HACCP system and helps distinguish between Key Food Safety Control Steps (KFSCS), where some are classified as CCPs, while others can be managed through Prerequisite Programs (PRPs) or Operational Prerequisite Programs (OPRPs). PRPs, including cleaning, sanitation, and employee hygiene practices, are implemented to control hazards at various stages, thereby reducing the need for all steps to be classified as CCPs. For further reference on food safety and hygiene practices, we consulted the GoAudit Food Safety and Hygiene Checklist platform, which provides practical guidelines for managing PRPs and CCPs in food service settings (GoAudit, 2023).

The workflow in the hospital begins from the receiving area, considered Critical Control Point No. 1 (CCP1), where all raw, canned, and frozen food materials are received from suppliers. The materials are examined and sorted in this area by the inspectors’ compliance with food safety requirements. The sorted items are stored in the storage rooms at appropriate temperatures for each item. The storage rooms, including warehouses and refrigerators, are considered Critical Control Point No. 2 (CCP2). Temperature, food quality, cleanliness, and insects are regularly monitored in the storage rooms. The chief chef requests food items from the storage room manager, which are then delivered to the kitchen areas for preparation. Red and white meat are cut and prepared in the meat cutting room, which is regarded as Critical Control Point No. 3 (CCP3). This area has knives and chopping boards. According to the HACCP color coding for cutting boards, they are colored differently: blue is for all marine creatures, yellow is for poultry, and red is for lamb and beef. The vegetable preparation and washing area, Critical Control Point No. 4 (CCP4), is responsible for washing and cutting vegetables and fruits for kitchen use or direct consumption. Green cutting boards are used, cleanliness is maintained, and insects are removed to prevent contamination of ready-to-eat foods. Cross-contamination is avoided by not using cutting boards from other areas, such as meat preparation. The main kitchen area is Critical Control Point No. 5 (CCP5), where all patient-served foods are cooked and monitored for temperature, and the correct cooking methods are followed to ensure safety. At Critical Control Point No. 6 (CCP6), the prepared meal is dispensed into the patient trays, and diet types are checked and ensured that hot food temperatures are above 60°C and cold food temperatures are 5°C and below. These trays are then transported to hospital wards for distribution to patients. This ensures proper nutrition and hygiene in the healthcare system (Saudi Food and Drug Authority, 2019). Carts with heating and cooling features are divided into hot and cold sections to maintain meal temperatures until patients arrive. The personal hygiene of food handlers, including cooks, nutritionists, food distributors, and cleaners, is monitored during all stages. They must also wear gloves and wash their hands multiple times before and after meals and rest periods. Hands are examined for wounds or damage, and a workflow chart in the nutrition department is created to facilitate sampling. The carts are designed to protect food handlers from respiratory or gastrointestinal infections, such as influenza and diarrhea.

### 2.3 Microbial analysis

#### 2.3.1 Preparing food samples

Twenty-five grams of each sample were weighed, placed in Stomacher bags, and mixed with 225 mL of sterile peptone water (0.1% BPW). The sample was then homogenized with peptone water for 5 min using a Stomacher mixer (Lab Blender 400, United Kingdom), and then 10 mL of the content of the Stomacher bags was taken to make the necessary dilutions for each analysis.

#### 2.3.2 Total count of aerobic bacteria

Twenty-eight grams of Nutrient Agar (Cat. No. CM0003, Oxoid Ltd., United Kingdom) was added to 1 L of distilled water and boiled at low heat. After that, it was sterilized in an autoclave at 121°C for 15 min. The agar was poured into sterile Petri dishes and left to solidify. One mL of dilution was spread on the Petri dish’s surface using a sterile plastic hockey stick. The petri dishes were incubated upside down in the incubator at 37°C for 48 h. The bacterial colonies were counted, and the total bacterial count was converted to the logarithm (Log 10 CFU g–1) (Yuliati et al., 2021).

#### 2.3.3 Total count of coliform bacteria

Thirty-nine grams of violet-red bile Agar (Cat. No. CM0107, Oxoid Ltd., United Kingdom) was added to 1 L of distilled water, boiled to dissolve completely, and poured into sterile Petri dishes. One mL of dilution was spread on the Petri dish’s surface using a sterile plastic hockey stick. The Petri dishes were incubated upside down at 37°C for 48 h. The presence of purple to pink colonies indicates the presence of coliform bacteria (Yuliati et al., 2021).

#### 2.3.4 Total number of fungi (yeasts and molds)

Thirty-nine grams of potato dextrose agar (Cat. No. CM0139, Oxoid Ltd., United Kingdom) was added to 1 L of distilled water and boiled at low heat. The agar was poured into sterile Petri dishes after autoclaving at 121°C for 15 min. 0.1 mL of dilution was taken and spread on the Petri dish’s surface using a sterile plastic hockey stick. The Petri dishes were incubated upside down in the incubator at 35°C for 2–3 days. The total number of fungi was counted, and the total fungi count was converted to the logarithm (Log 10 CFU g–1) (Damena et al., 2022).

#### 2.3.5 Detection of *Salmonella*

Two media, lactose broth (Cat. No. CM0137, Oxoid Ltd., UK) and hektoen enteric agar (Cat. No. CM0419, Oxoid Ltd., United Kingdom), were prepared separately. Both media were sterilized in an autoclave at 121°C for 15 min. The sterilized contents were transferred into sterile Petri dishes. Twenty-five grams of the specimen was added to 225 mL lactose broth in stock maker bags and mixed for 2 min. The sample was incubated at 37°C for 24 h. The presence of *Salmonella* was indicated by blue/green colonies with shiny black centers on HE medium and bright black-centered or red/pink colonies on XLD medium (Younus et al., 2020).

#### 2.3.6 Detection of *Listeria monocytogenes*

A volume of 0.5 mL of dilution was poured on Petri dishes containing Listeria selective agar base medium and the Listeria selective supplement. The Petri dishes were incubated at 37°C for 48 h. Greenish-black colonies indicated the presence of *L. monocytogenes* (Yehia et al., 2016).

### 2.4 Chemical analysis

Twenty grams of each sample was used to determine mycotoxins, heavy metals, and pesticide residues.

#### 2.4.1 Determination of mycotoxins

Mycotoxins were determined using high performance liquid chromatography-spectrofluorometric detection (HPLC-FLD, Thermo Fisher Scientific Inc. Waltham, Massachusetts, United States) device according to the EN ISO 16050:2011 method (Aguilera-Luiz et al., 2011).

#### 2.4.2 Determination of heavy metals

Nickel (Ni), tin (Sn), arsenic (As), cadmium (Cd), lead (Pb), and mercury (Hg) were determined by inductively coupled plasma mass spectrometry (ICP-MS; Thermo Fisher Scientific Inc., Waltham, MA, USA) in accordance with EN 15763 (2009a, 2009b), EN 13805 (2002), AFNOR-NF EN 13804 (2002), and EN ISO 17294 (2003).

#### 2.4.3 Determination of pesticide residues

Pesticide residues were determined using gas chromatography-tandem mass spectrometry (GC-MS/MS, Thermo Fisher Scientific Inc. Waltham, Massachusetts, United States) and liquid chromatography–tandem mass spectrometry (LC-MS/MS, Thermo Fisher Scientific Inc. Waltham, Massachusetts, United States) devices according to the EN 15662:2009-02 (Din, 2008) method.

### 2.5 Detection of physical hazards

Physical hazards such as metal, hair, glass, plastic, and insects were observed through visual observation. A form was created for observation during sampling and swabbing according to the identified CCPs.

### 2.6 Monitoring the temperatures of patients’ meals

After cooking, the temperatures were recorded using a food thermometer and an infrared thermometer (Electronic Temperature Instruments Ltd., West Sussex, United Kingdom). Food preservation trolleys, equipped with both cooling and heating features, were employed to monitor and maintain food temperatures from production to delivery. Despite this, significant temperature fluctuations were observed during transport. To mitigate this, it is essential to implement better thermal insulation in food delivery trolleys and continuously monitor food temperatures to ensure they remain within the recommended ranges. These strategies will help prevent microbial growth and maintain food safety. Temperatures were recorded on a designated form during sampling to ensure accuracy.

### 2.7 Ethical considerations

This study adhered strictly to the ethical standards set by the Ministry of Health, Kingdom of Saudi Arabia. Ethical approval was obtained from the Scientific Research Ethics Committee, Deanship of Scientific Research, King Faisal University (certificate No. KFU-NEC-2021-DEC-EA000Z100). The study followed established protocols to ensure participants’ safety, confidentiality, and the integrity of the hospital institution while handling food samples from hospital kitchens. These measures, in addition to the initial ethical review, reinforce our commitment to scientific integrity and ethical responsibility, ensuring the protection of both researchers and participants throughout the study.

### 2.8 Risk assessment models

**Figure 1:**
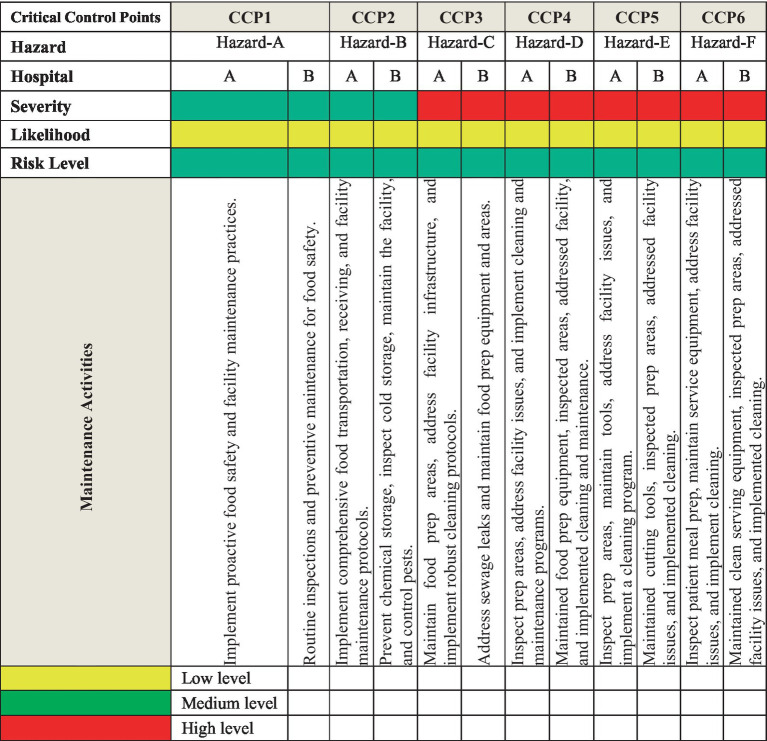
Comprehensive analysis of risk assessment matrix of physical risks in hospital environments and recommended control measures across hospitals in Al-hasa, Saudi Arabia. Hazard-A, Raw food contamination, facility infrastructure issues. Hazard-B, Freezer/cooler contamination, facility infrastructure flaws. Hazard-C, Food preparation area contamination risks. Hazard-D, Prepared food contamination and facility maintenance problems. Hazard-E, Prepared food contamination facility hygiene issues. Hazard-F, Patients’ meal contamination and facility equipment issues.

**Figure 2:**
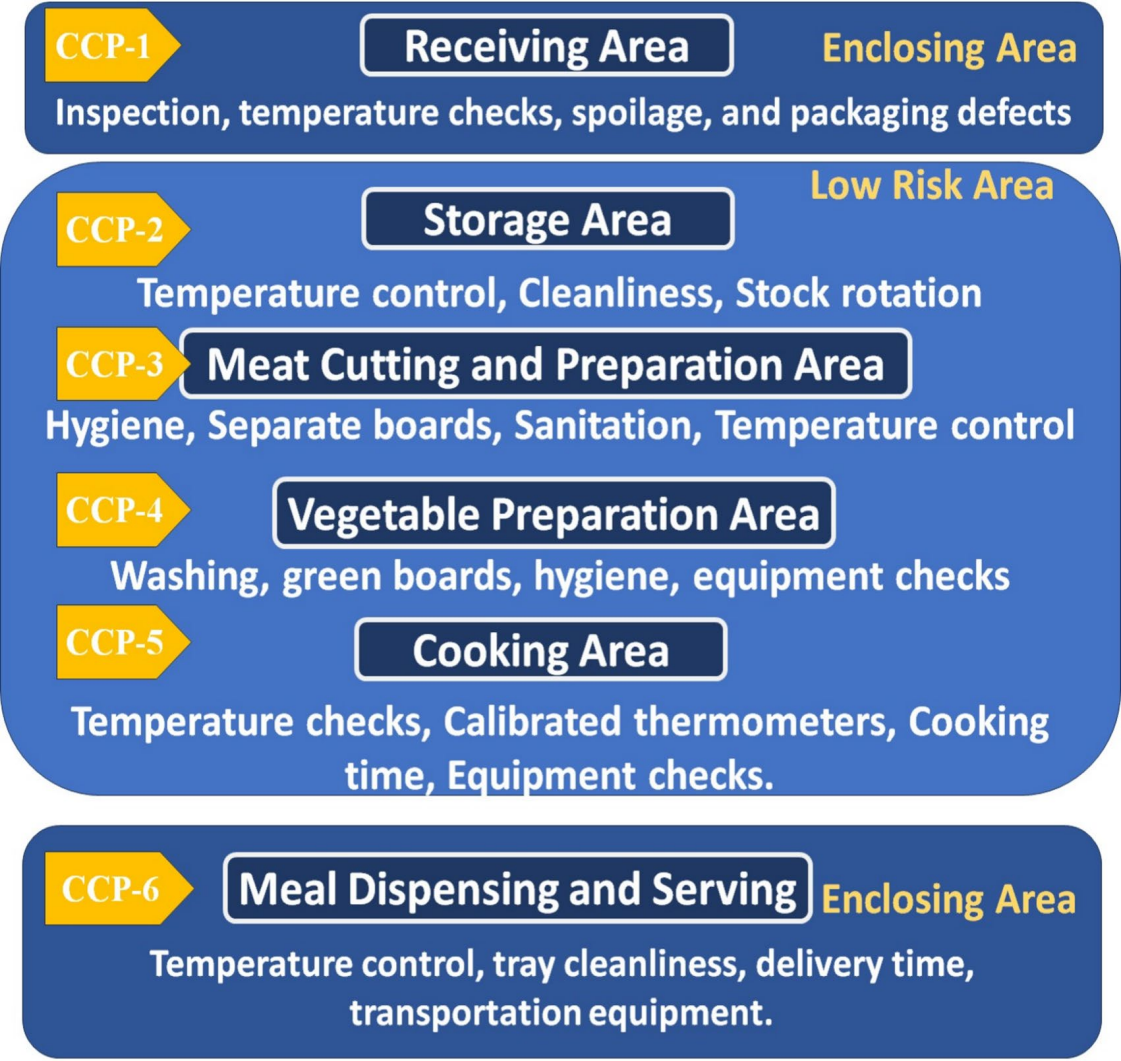
Flowchart representing the HACCP inspection process for hospitals, with six critical control points (CCPs) and monitoring steps.

Within the food service chains of hospital nutrition departments, various risk assessment models are employed to ensure food safety and quality assurance. Qualitative and quantitative models are commonly used to identify potential hazards and control measures. Hazard Analysis and Critical Control Points (HACCP) and Failure Mode and Effects Analysis (FMEA) were used to find possible problems in food processing. Risk assessment matrix models are implemented to prioritize risks and evaluate potential hazards across six Critical Control Points (CCPs) in hospital food preparation and service environments. Each hazard (labeled in Figure 1 as Hazard A to F) is assessed according to its severity and likelihood, which calculates the overall risk level. The risk levels range from low to high, indicating each hazard’s potential danger (Teffo and Tabit, 2020). Severity refers to the potential impact of a hazard on food safety or health outcomes. Likelihood evaluates how often the hazards might occur. Risk level is determined by combining the severity and likelihood ratings, guiding the prioritization of control measures. The matrix also outlines maintenance activities for each CCP to mitigate identified risks. These activities range from routine inspections, facility maintenance, and pest control to addressing infrastructure issues in food preparation and service areas. The matrix is a practical tool for guiding hospital decision-making to ensure food safety, as shown in Figure 2.

### 2.9 Statistical analysis

The data was analyzed using the SPSS statistics program, version 29 (IBM, New York, United States). The same program was used to calculate the mean and standard deviation. The Levene test verified the homogeneity of variance (Levene, 1960). Differences between hospital variables were statistically analyzed using the Mann–Whitney test (Test, 2000) for two independent samples. Differences between samples (before and after) of the same hospital were statistically analyzed using a paired-sample T-test. Parametric data were statistically analyzed using one-way analysis of variance (ANOVA) using the SPSS program. In contrast, the difference between means was determined using the Duncan Multiple Range Test at a 5% probability level (*p* ≤ 0.05). The heatmap was constructed using R programming, version R 4.3.2. (R Core Development Team, The R Foundation, Vienna, Austria).

## 3 Results and discussion

### 3.1 Microbial analyses

The high Total Bacterial Count (TBC) for raw meat, chicken, and fish, reaching up to 5 × 10^6^ log cfu/g, suggests significant microbial contamination. Although these values are within the acceptable limits as per GSO and CODEX standards, they still represent a considerable microbial presence that could lead to potential risks if not properly managed. High bacterial loads in meat and chicken indicate that these raw materials are particularly susceptible to contamination during critical stages such as handling, storage, and transportation. These findings stress the importance of improving hygiene practices and monitoring across these stages to mitigate risks of contamination. The presence of coliforms in these samples further highlights the risk of fecal contamination, a key food safety concern. Coliform bacteria, especially in raw meats, suggest lapses in hygiene during handling, such as improper washing or contamination from untreated water sources. This emphasizes the importance of stringent sanitation protocols at each stage of food processing to prevent such contamination from reaching hospital patients. Moreover, while the absence of *Salmonella* and *Listeria monocytogenes* in most samples is a positive finding, indicating effective pathogen control, the coliforms’ presence points to areas where food safety practices need to be reinforced, particularly during the early stages of food handling.

While the absence of *Salmonella* and *Listeria monocytogenes* in most samples is a positive finding, it also suggests that pathogen control measures, such as temperature control and proper sanitation, are well-managed in most stages of the food service chain. These findings should be interpreted cautiously, however, as high bacterial loads in raw meat and chicken samples still present a risk for secondary contamination, particularly if food handling or storage conditions are compromised at any point. Although the TBC in fish samples is slightly lower than in meat and chicken, it still reaches significant levels, with values up to 1 × 10^6^ log cfu/g. The presence of coliforms at relatively low levels (5 × 10^4^ log cfu/g) in fish samples suggests that similar contamination risks are present across different food categories, including fish. This finding indicates that cross-contamination between raw meats and fish could occur if not properly managed, highlighting the need for effective segregation and thorough washing practices during food preparation. Again, the absence of *Salmonella* and *Listeria monocytogenes* in these samples indicates effective pathogen control measures during handling and storage (Kuehn et al., 2016). The Total Mold and Yeast (TMY) count was primarily observed in bakery products and prepared meals such as cooked vegetables and fish. The relatively lower TMY counts in bakery products (1 × 10^4^ log cfu/g) can be attributed to the lower moisture content, which inhibits fungal growth. However, the presence of molds in bakery products indicates that storage conditions may still contribute to fungal contamination, emphasizing the need for adequate moisture control and regular monitoring of storage conditions to prevent mold proliferation and mycotoxin production. The detection of coliforms in cucumber fruits and bakery products, even within acceptable limits, reinforces the need for rigorous washing and handling practices to prevent contamination. While these levels do not exceed safety limits, their presence underscores the potential for cross-contamination if adequate hygiene is not maintained. This finding highlights a gap in food handler education and the importance of strict sanitation during the washing and preparation of ready-to-eat foods. Prepared meals, such as soups, cooked vegetables, fresh salads, cooked rice, and cooked fish, typically exhibited lower microbial loads than raw materials. This reduction in microbial load suggests that cooking plays an effective role in reducing microbial contamination. However, the presence of some microbes in these meals, such as coliforms and molds, even at low levels, suggests post-cooking contamination. This could occur during handling, storage, or serving before the food reaches patients. Such findings point to areas where stricter hygiene measures are needed post-cooking, especially for foods that are not consumed immediately after preparation.

The TBC in cooked vegetables and fish is significantly lower than in raw materials, with values around 1 × 10^5^ log cfu/g. This reduction in microbial loads reflects the effectiveness of cooking. However, the presence of TMY and coliforms in these samples, though minimal, suggests that post-cooking contamination might occur, possibly during handling or storage before serving (Munir et al., 2020). Fresh salads and cooked rice exhibited higher TBC (up to 1 × 10^6^ log cfu/g), likely due to the minimal processing involved. Fresh salads, in particular, are prone to microbial contamination if not properly washed and handled, as indicated by the presence of coliforms. These findings highlight the importance of strict hygiene protocols to prepare minimally processed foods (Shahbaz et al., 2022). The lower microbial loads in soups and bakery products, with TBC values around 1 × 10^5^ log cfu/g, demonstrate that high temperatures during preparation, cooking, and baking are effective at killing most microbes. The absence of TMY and coliforms in these samples indicates that these food items are less prone to contamination, thanks to the protective effect of heat treatment. However, this should not lead to complacency, as improper handling or storage after preparation could still lead to microbial growth and contamination.

The data in Supplementary Table S1 reveal significant microbial contamination in raw meat samples. For instance, in Hospital A (HA), the TBC for raw meat ranged from 2.48 to 3.91 log cfu/g, while in Hospital B (HB), it ranged from 2.25 to 3.79 log cfu/g. These values suggest that although both hospitals maintain some degree of microbial control, there are still considerable risks, significantly when the TBC exceeds 3 log cfu/g, a threshold often associated with increased risk of spoilage and potential foodborne illness (Okoh et al., 2019; Kim et al., 2024). This level of contamination is concerning as it indicates potential lapses in raw meat’s handling and storage practices, which could lead to foodborne illnesses if not adequately addressed (Barad et al., 2020). Moreover, coliform bacteria in raw meat, with counts ranging from 1.59 to 2.96 log cfu/g, indicates possible fecal contamination, a serious concern for food safety. The detection of Staphylococcus spp. and *Listeria monocytogenes*, although in lower concentrations (generally below detection limits), is particularly troubling given their pathogenic potential, especially in hospital settings where patients may have compromised immune systems (Musa et al., 2020).

The microbial load in raw vegetables also showed considerable variation. The TBC in raw vegetables at HA ranged from 2.45 to 3.07 log cfu/g; at HB, it ranged from 2.62 to 3.25 log cfu/g. The study suggests that vegetables, similar to meat, can be highly contaminated due to improper handling or insufficient washing (Oie et al., 2008). The relatively high coliform counts in vegetables (up to 3.06 log cfu/g) further emphasize the need for better sanitation practices during vegetable processing. The presence of coliform bacteria, with counts ranging from 1.50 to 3.06 log cfu/g in raw vegetables, further underscores the need for stringent hygiene practices during preparing and storing these materials (Chinakwe et al., 2022). Detecting molds and yeasts in several samples, particularly in bakery products, also indicates inadequate storage conditions that may facilitate fungal growth, potentially leading to mycotoxin production (Al-Musawi et al., 2023).

For implications for food safety, the microbial analysis results from Table 1 indicate that while most food items are within acceptable microbial limits according to GSO and CODEX standards, there are still areas of concern that need to be addressed to ensure food safety in hospital settings: (1) Enhanced hygiene practices: The presence of coliforms in both raw materials and prepared meals suggests lapses in hygiene practices, particularly during handling and storage, so, hospitals should reinforce sanitation protocols, primarily where raw and cooked foods are handled (Singh et al., 2014). (2) Improved storage conditions: High microbial loads in raw materials, particularly meat, and vegetables, highlight the need for better storage conditions; controlling temperature and humidity levels can help reduce microbial growth and prevent spoilage (Adams et al., 2024). (3) Regular monitoring and testing: Continuous microbial testing is crucial for early contamination detection and ensuring that microbial loads remain within safe limits; this is particularly important for raw materials more prone to contamination (Song et al., 2019). (4) Adherence to HACCP Principles: These findings emphasize the importance of adhering to HACCP protocols; hospitals can mitigate microbial contamination risks by identifying and managing CCPs throughout the food service chain (Angelillo et al., 2001).

**Table 1:** Microbial profile (log × 10 cfu/g) of studied food samples according to GSO and CODEX standards.

Food items		Microbial loads (log ×10 cfu/g)				
		TBC	TMY	TC	SS	LM
Raw materials for meal	Meat	^*^1 × 10^6^	^***^NA	^***^1 × 10^2^	*	^***^ND/25 g
					^**^ND/25 g	
		^***^1 × 10^6^			^***^ND/25 g	
	Chicken	^*^5 × 10^6^	^***^NA	^***^1 × 10^2^	*	^***^ND/25 g
		^***^1 × 10^6^			^***^ND/25 g	
	Fish	^*^1 × 10^6^	^***^NA	^***^5 × 10	^***^ND/25 g	^***^ND/25 g
		^***^5 × 10^5^		^**^5 × 10^2^		
	Fresh egg	-	-	-	*	-
					^**^ND/25 g	
					^***^ND/25 g	
	Bakery	-	^*^1 × 10^4^	-	-	-
	Cucumber fruits	^***^1 × 10^6^	-	^***^1 × 10^2^	*	*
				^*^1 × 10^2^		
Prepared meal	Soup*	^*^1 × 10^5^	-	-	-	-
	Cooked vegetables	^***^1 × 10^5^	^***^1 × 10	^***^1 × 10	^***^ND/25 g	^***^ND/25 g
	Fresh salad	^***^1 × 10^6^	-	^***^1 × 10^2^	*	*
				^*^1 × 10^2^		
	Cooked rice	^*^1 × 10^6^	-	^*^1 × 10^2^	*	-
	Cooked fish	^***^1 × 10^5^	^***^1 × 10	^***^1 × 10	^***^ND/25 g	^***^ND/25 g
	Bakery	^*^1 × 10^5^	-	-	*	*

TBC, total bacterial count; TMY, total mold and yeast; TC, total Coliform spp.; SS, Salmonella spp.; LM, *Listeria monocytogenes*; NA, Not available; ND, Not detected. ^*^GSO, ^**^CODEX, and ^***^ISO.

**Figure 3:**
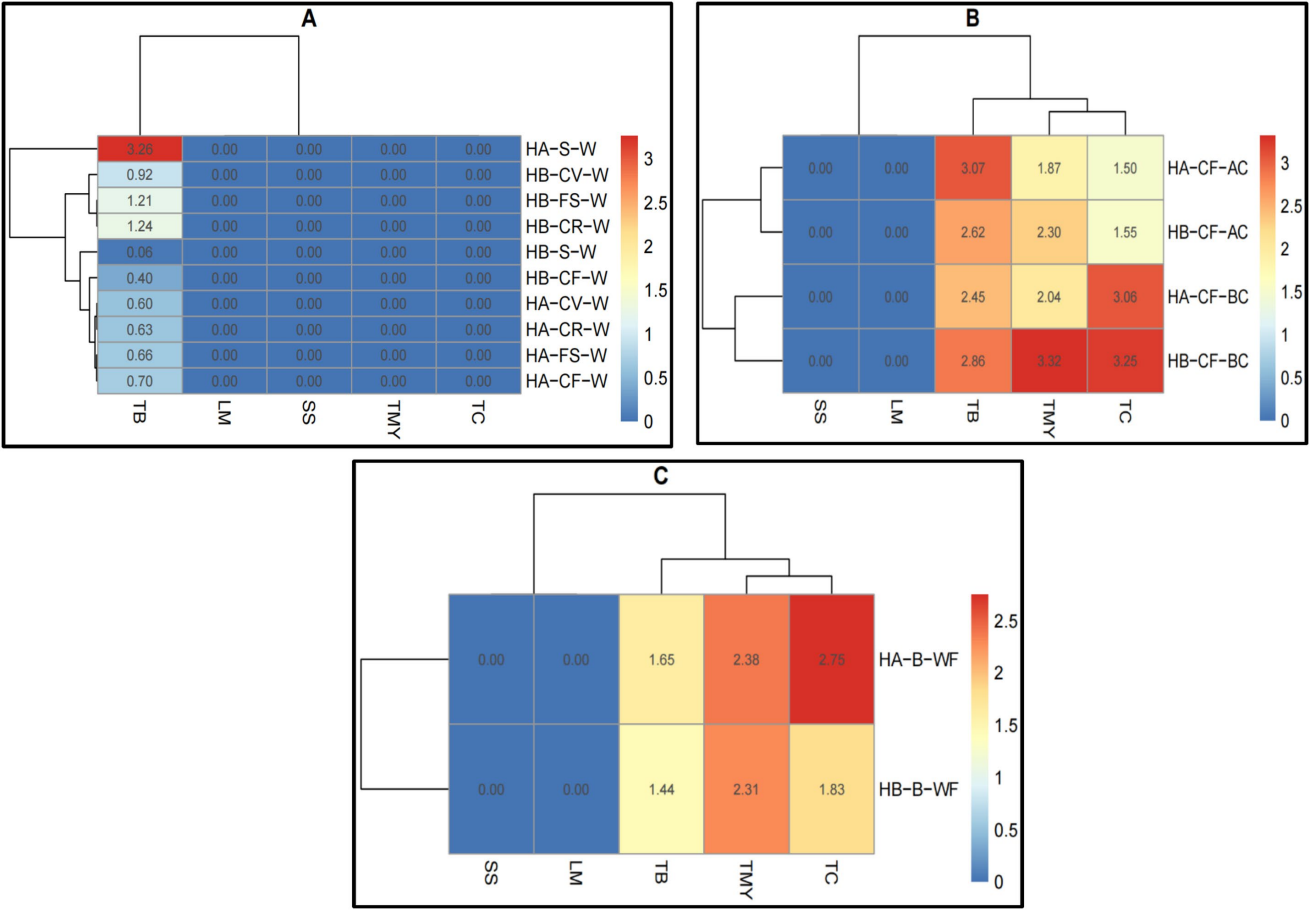
Heatmaps of microbial load (log ×10 cfu/g) analysis of **(A)** raw meat, **(B)** raw vegetable, and **(C)** bakery materials (wheat flour) in hospitals of Al-Hasa Governorate, Saudi Arabia. HA, Hospital A; HB, Hospital B; TB, Total Bacterial Count; TMY, Total Mold and Yeast; TC, Total Coliform; SS, Staphylococcus spp.; LM, *Listeria monocytogenes*; M, Meat; C, Chicken; F, Fish; E, Fresh Egg; B, Bakery; WF, Wheat Flour; W, Whole Sample; S, Surface sample; CF, Cucumber Fruit; BC, Before Cutting; AC, After Cutting.

Both hospitals did not detect The SS and LM in raw meat, raw vegetables, or bakery materials (Figures 3A–C; Supplementary Table S2). The TB was higher in meat and fish samples in both hospitals, while the TMY was higher in meat, fish, and chicken samples (Figure 3A). There is no apparent difference between the whole and surface samples. Hospital A’s samples showed higher TB after cutting the cucumber fruit, while Hospital B’s samples showed higher TMY and TC before cutting the cucumber fruit (Figure 3B). The TMY was higher in bakery (wheat flour) samples from both hospitals, whereas the TC was higher in hospital A samples (Figure 3C). These results illustrate the overall microbial load across different food types, demonstrating that specific food categories, such as raw meats, exhibit higher contamination levels than others (Mahato, 2019).

**Figure 4:**
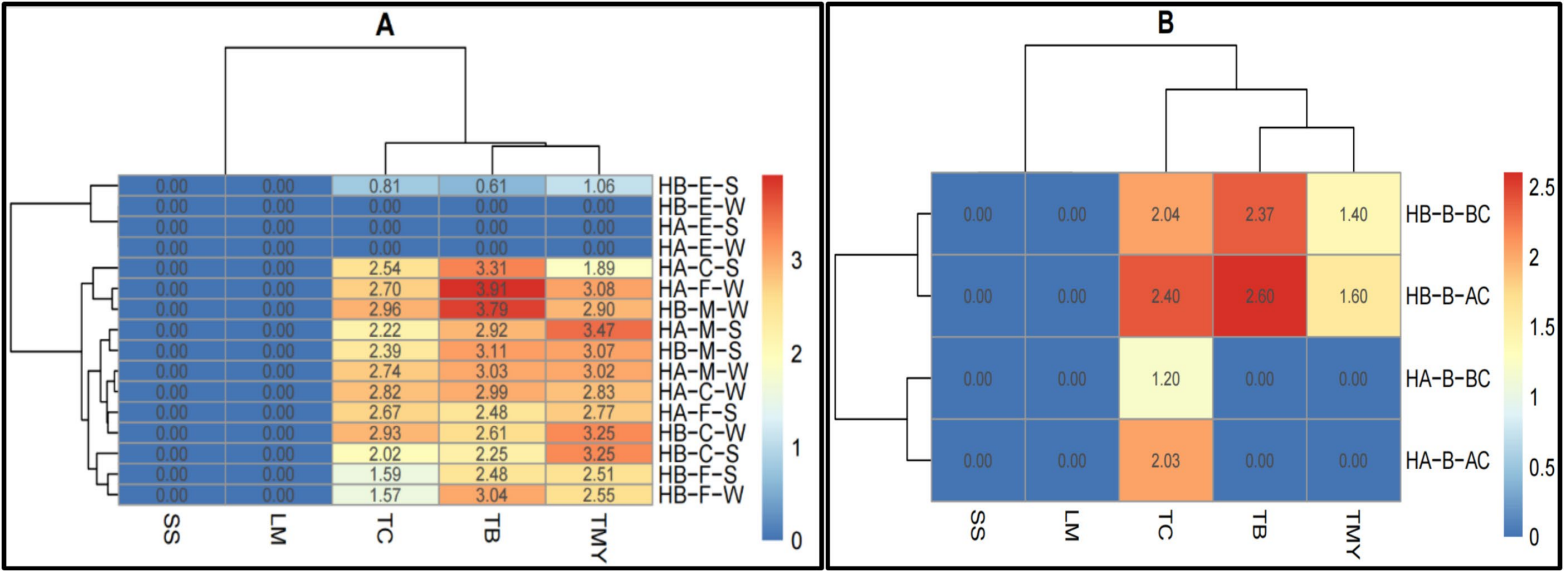
Heatmaps of comparative microbial profiles (log ×10 cfu/g) of **(A)** meal preparation and **(B)** bakery preparation areas in hospitals of Al-Hasa Governorate, Saudi Arabia. HA, Hospital A; HB, Hospital B; TB, Total Bacterial Count; TMY, Total Mold and Yeast; TC, Total Coliform; SS, Staphylococcus spp.; LM, *Listeria monocytogenes*; S, Soup; CV, Cooked vegetables; FS, Fresh salad; CR, Cooked Rice; CF, Cooked Fish; W, Whole sample; B, Bakery (wheat flour); BC, Before Cutting; AC, After Cutting.

The results presented in Figure 4 visualize the microbial profiles of the studied food samples in the meal preparation (Figure 4A) and bakery preparation (Figure 4B) areas (Supplementary Table S2). TC, TMY, SS, and LM were not counted in the meal preparation area. However, the TB count in Hospital A’s soup was higher. Both hospitals counted the TB in cooked vegetables, fresh salad, cooked rice, and cooked fish, but it was significantly lower than in the soup samples in hospital A (Figure 4A). Both hospitals’ bakery preparation areas showed no traces of SS or LM. However, TC was higher in both hospitals’ bakery preparation areas, whereas TB and TMY were higher in hospital B (Figure 4B). These results show the microbial contamination levels at various CCPs, reinforcing the need for enhanced hygiene practices at critical stages such as meat cutting and vegetable preparation (Bosede Fasoyiro et al., 2010).

**Figure 5:**
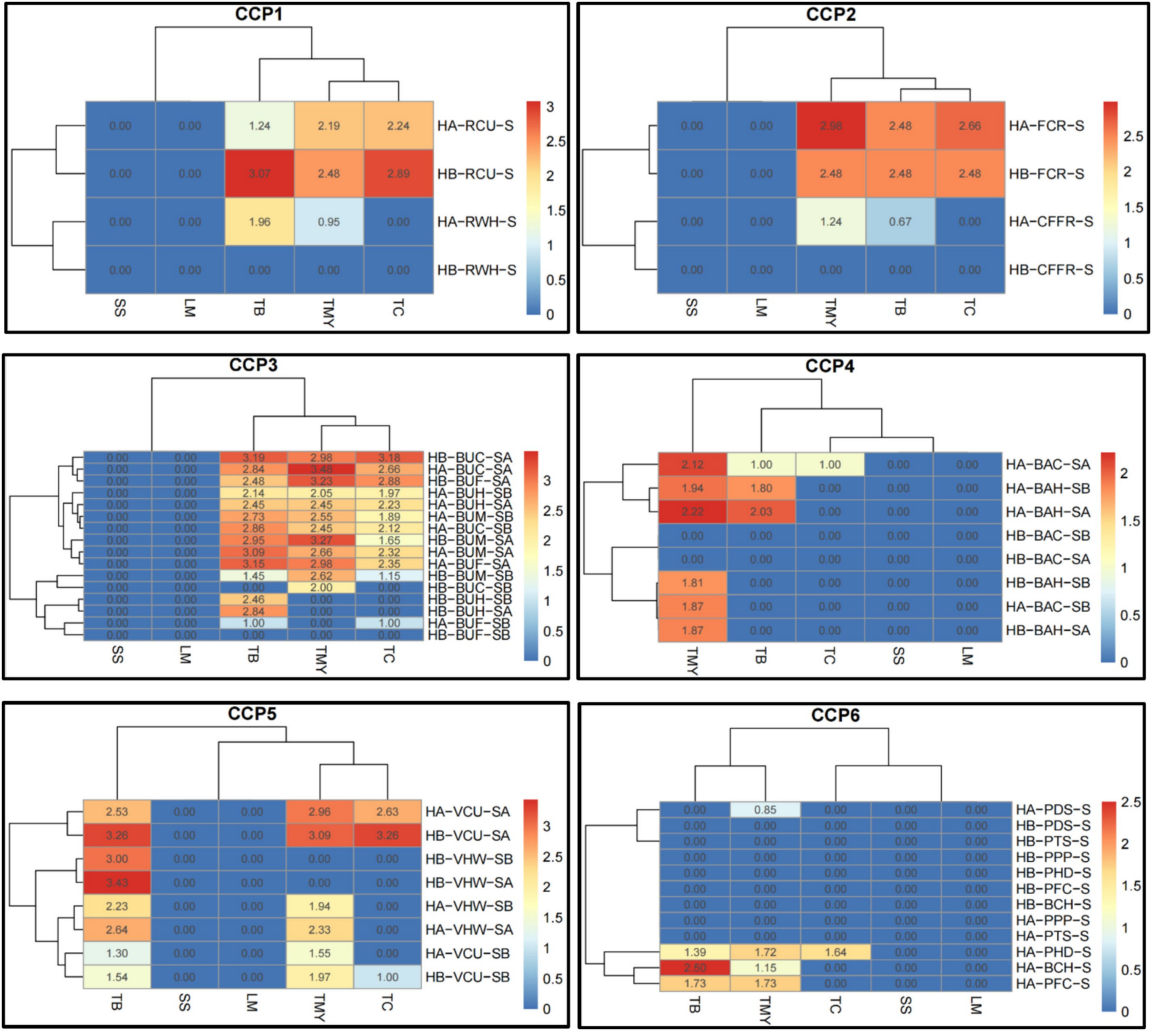
Heatmaps of comparative microbial loads (log ×10 cfu/g) in critical control points (CCP) across hospitals in Al-Hasa Governorate, Saudi Arabia. HA, Hospital A; HB, Hospital B; TB, Total Bacterial Count; TMY, Total Mold and Yeast; TC, Total Coliform; SS, Staphylococcus spp.; LM, *Listeria monocytogenes*; RWH, Receiving worker’s hand; RCU, Containers before unloading; S, Swap; FCR, From the cold room; CFFR, Container from the freezer room; BUH, Butchers’ hands; BUM, Meat cutting board; BUC, Chicken cutting board; BUF, Fish cutting board; SB, Swap Before use; SA, Swap After use; BAH, Baker’s hands; BAC, Bakker’s cutting boards; VCU, Vegetable cutting board; VHW, Hands of vegetable preparation workers; PDS, Patient dish before serving; PPP, Plastic spoons for patients; PHD, Hands of food distributor; BCH, Cook’s hands; CCP1, Critical control point of receiving area; CCP2, Freezer and cooling rooms; CCP3, Butchery area; CCP4, Bakery preparation area; CCP5, Vegetable and salad preparation area; and CCP6, Patient meal dispensing area.

The data presented in Figure 5 reveals the distribution of microbial contamination across various food materials and CCPs, aiding in identifying areas where microbial loads exceed safe limits and guiding targeted interventions (Supplementary Table S3). The CCP1 microbial data in the matrix range from 0.00 to 3.07. The samples were taken from the worker’s hand (RWH) and containers before unloading (RCU). The SS and LM were not detected in both hospitals from both sample points. The TC, TMY, and TB were also not detected in RWH in hospital B and TC in hospital A in the same sample area. The TB, TC, and TMY were higher in Hospital B in RCU area. The CCP2 microbial data in the heatmap range from 0.00 to 2.98. The samples were taken from the cold room (FCR) and containers from the freezer room (CFFR). The SS and LM were not detected in both hospitals from both sample points. The TC, TMY, and TB were also not detected in CFFR in hospital B and TC in hospital A in the same sample area. The TB, TC, and TMY were higher in both hospitals in FCR area.

The CCP3 microbial data range from 0.00 to 3.48. The samples were taken from butchers’ hands (BUH), meat cutting board (BUM), chicken cutting boards (BUC), and fish cutting boards (BUF). The sample swaps were taken before (SB) and after (SA) use. None of the samples from all sample points had the SS and LM in both hospitals. Similarly, the TB and TC were not detected in HB-BUC-SB; TC, TB, and TMY in HB-BUF-SB; TMY in HB-BUH-SB, HB-BUH-SA, and HA-BUF-SB; and TC in HB-BUH-SB and HB-BUH-SA. The TB was higher in HB-BUC-SA, followed by HA-BUF-SA. The TMY was higher in HA-BUC-SA, followed by HB-BUM-SA, and the TC was higher in HB-BUC-SA, followed by HB-BUF-SA. The CCP4 microbial data in the heatmap range from 0.00 to 2.22. The samples were taken from baker’s hands (BAH) and baker’s cutting boards (BAC). The sample swaps were taken before (SB) and after (SA) use. The SS and LM were not detected in both hospitals from both sample points before and after use. Other than HA-BAC-SA, none of the treatment combinations had TC. The TB was only detected in HA-BAC-SA, HA-BAH-SA, and HA-BAH-SB. The TMY was not found in HB-BAC-SA and HA-BAC-SB. However, it was higher in HA-BAH-SA, followed by HA-BAC-SA.

The CCP5 microbial data range from 0.00 to 3.43. The samples were taken from a vegetable cutting board (VCU) and the hands of vegetable preparation workers (VHW). The sample swaps were taken before (SB) and after (SA) use. The SS and LM were not detected in both hospitals from both sample points before and after use. The TC was detected only in HB-VCU-SA, HA-VCU-SA, and HB-VCU-SB. The TB was higher in HB-VHW-SA, followed by HB-VCU-SA, whereas the TMY was found in HB-VCU-SA and HA-VCU-SA. The CCP6 microbial data range from 0.00 to 2.50. The samples were taken from patient dishes before serving (PDS), plastic spoons for patients (PPP), hands of food distributor (PHD), and cook’s hands (BCH). The SS and LM were not detected in both hospitals from all sample points. A similar trend was observed in TC, except it was found only in HA-PHD-S. Overall, the highest count of TB was determined in HA-BCH-S, followed by HA-PFC-S and HA-PHD-S. The TMY was detected only in HA-PFC-S, followed by HA-BCH-S, and HA-PHD-S.

Identifying Critical Control Points (CCPs) in the food service chain is crucial for preventing microbial contamination. The results indicate that while some CCPs were effectively managed, others exhibited higher microbial loads, particularly those related to preparing and handling raw meat and vegetables (Ali et al., 2022; Uzoigwe and Kongolo, 2024). The microbial analysis of swab samples from the meat cutting areas (CCP3) showed that TBC ranged from 2.14 to 3.19 log cfu/g, indicating possible cross-contamination risks if proper hygiene protocols are not followed (Miner et al., 2020). The study also highlights the importance of temperature control as a CCP. The results indicate that the temperature of hot food often decreased to levels that could potentially allow bacterial growth before reaching the patient’s table, while cold food samples were not consistently maintained at the required temperature set by the Saudi Food and Drug Authority (SFDA) (Politis et al., 2017). These findings emphasize the need for improved temperature monitoring systems to ensure that food remains safe from production to consumption (De Jonge et al., 2004; Abass et al., 2024).

The results presented emphasize the importance of adhering to proper food safety protocols throughout the food service chain in hospitals. While some Critical Control Points (CCPs) are well-managed, others, particularly raw meat and vegetable preparation, require further attention to prevent microbial contamination. HACCP protocols, temperature control, and strict hygiene practices are essential to ensure that food safety is maintained from preparation through to patient consumption.

The microbial analysis results significantly affect food safety within the hospitals studied. Despite implementing HACCP principles, microbial contamination at critical stages of the food service chain suggests gaps in the current food safety management systems (AlNaim et al., 2024). These gaps may arise from inadequate training of food handlers, lapses in hygiene practices, or insufficient monitoring of CCPs. To address these issues, it is recommended that hospitals reinforce their HACCP protocols by conducting regular audits, providing ongoing training to staff, and enhancing their temperature monitoring systems. Additionally, strict control measures should be implemented at identified high-risk areas, such as raw meat and vegetable preparation zones, to prevent cross-contamination and ensure that food served to patients is safe for consumption (Angelillo et al., 2001; Trafialek and Kolanowski, 2017).

### 3.2 Chemical analyses

The chemical analysis results in Tables 2–4 indicate the study’s analytical results. The data presented in Table 2 focus on detecting aflatoxin subtypes (B1, B2, G1, and G2) in wheat flour samples collected from two hospital facilities (Hospital A and Hospital B). We found that the levels of aflatoxin B1, B2, G1, and G2 in all examined wheat flour samples were below the respective limits of Detection (LOD) and limits of quantification (LOQ) established for this study. Specifically, the LODs were 0.005 μg/kg, 0.05 μg/kg, 0.01 μg/kg, and 0.01 μg/kg for aflatoxin B1, B2, G1, and G2, respectively. The corresponding LOQs were 0.01 μg/kg, 0.10 μg/kg, 0.05 μg/kg, and 0.05 μg/kg, respectively. The values were found to be below the detection and quantification thresholds, indicating the absence of contamination with these mycotoxins. It indicated that all individual aflatoxin subtypes were below the designated LODs; the total aflatoxin content was also considered not applicable (NA) for the analyzed wheat flour samples from both hospitals (Zahra, 2019; Abdu et al., 2024).

**Table 2:** Detection of aflatoxin types (B1, B2, G1, G2) contamination in wheat flour samples from the kitchens of two hospitals.

Samples	Type of mycotoxin	Hospital A		Hospital B		LOD	LOQ
		1st sample	2nd sample	1st sample	2nd sample		
Wheat flour	Aflatoxin B1	ND	ND	ND	ND	0.005	0.01
	Aflatoxin B2	ND	ND	ND	ND	0.05	0.10
	Aflatoxin G1	ND	ND	ND	ND	0.01	0.05
	Aflatoxin G2	ND	ND	ND	ND	0.01	0.05
	Total aflatoxins	NA	NA	NA	NA	NA	NA

The unit of mycotoxins measurement is micrograms μg/kg. ND, not detected; NA, Not Applicable; LOD, Limit of Detection; LOQ, Limit of Quantification (the considered limit).

**Table 3:** Levels of heavy metals concentration (mg/kg) in fresh raw materials and cooked patients’ meals from two hospitals.

Samples	Heavy metals	Hospital A		Hospital B		LOD	LOQ
		1st sample	2nd sample	1st sample	2nd sample		
Fresh raw materials							
Vegetable (Cucumber) before washing	Arsenic (As)	ND	<LOQ	<LOQ	<LOQ	0.019	0.066
	Cadmium (Cd)	ND	ND	ND	ND	0.017	0.059
	Lead (Pb)	ND	ND	ND	ND	0.006	0.022
	Mercury (Hg)	ND	ND	ND	ND	0.014	0.047
	Nickel (Ni)	<LOQ	<LOQ	<LOQ	<LOQ	0.028	0.095
	Stannum Tin (Sn)	ND	ND	ND	ND	0.014	0.047
Vegetable (Cucumber) after washing	Arsenic (As)	ND	ND	ND	ND	0.019	0.066
	Cadmium (Cd)	ND	ND	ND	ND	0.017	0.059
	Lead (Pb)	ND	ND	ND	ND	0.006	0.022
	Mercury (Hg)	ND	ND	ND	ND	0.014	0.047
	Nickel (Ni)	<LOQ	<LOQ	<LOQ	<LOQ	0.028	0.095
	Stannum Tin (Sn)	ND	ND	ND	ND	0.014	0.047
Wheat flour	Arsenic (As)	ND	ND	ND	ND	0.019	0.066
	Cadmium (Cd)	ND	<LOQ	<LOQ	ND	0.017	0.059
	Lead (Pb)	ND	ND	ND	ND	0.006	0.022
	Mercury (Hg)	ND	ND	ND	ND	0.014	0.047
	Nickel (Ni)	0.2	0.22	0.12	0.24	0.028	0.095
	Stannum Tin (Sn)	ND	ND	ND	ND	0.014	0.047
Chicken	Arsenic (As)	<LOQ	ND	ND	ND	0.019	0.066
	Cadmium (Cd)	ND	ND	ND	ND	0.017	0.059
	Lead (Pb)	<LOQ	0.02	<LOQ	0.09	0.006	0.022
	Mercury (Hg)	0.09	<LOQ	<LOQ	ND	0.014	0.047
	Nickel (Ni)	0.11	<LOQ	<LOQ	0.68	0.028	0.095
	Stannum Tin (Sn)	ND	ND	ND	ND	0.014	0.047
Meat	Arsenic (As)	<LOQ	ND	ND	ND	0.019	0.066
	Cadmium (Cd)	ND	ND	ND	ND	0.017	0.059
	Lead (Pb)	0.03	ND	<LOQ	<LOQ	0.006	0.022
	Mercury (Hg)	ND	ND	ND	ND	0.014	0.047
	Nickel (Ni)	0.36	<LOQ	0.20	<LOQ	0.028	0.095
	Stannum Tin (Sn)	ND	ND	ND	ND	0.014	0.047
Fish	Arsenic (As)	1.61	1.31	0.46	0.5	0.019	0.066
	Cadmium (Cd)	ND	ND	ND	ND	0.017	0.059
	Lead (Pb)	ND	<LOQ	ND	0.25	0.006	0.022
	Mercury (Hg)	0.05	0.07	0.06	0.05	0.014	0.047
	Nickel (Ni)	<LOQ	< LOQ	<LOQ	<LOQ	0.028	0.095
	Stannum Tin (Sn)	ND	ND	ND	ND	0.014	0.047
Egg (Egg shell outer surface)	Arsenic (As)	ND	ND	< LOQ	ND	0.019	0.066
	Cadmium (Cd)	ND	ND	ND	ND	0.017	0.059
	Lead (Pb)	<LOQ	ND	<LOQ	<LOQ	0.006	0.022
	Mercury (Hg)	ND	ND	ND	ND	0.014	0.047
	Nickel (Ni)	0.32	0.36	0.14	0.29	0.028	0.095
	Stannum Tin (Sn)	ND	ND	ND	ND	0.014	0.047
Egg (Internal contents)	Arsenic (As)	<LOQ	ND	<LOQ	ND	0.019	0.066
	Cadmium (Cd)	ND	<LOQ	<LOQ	ND	0.017	0.059
	Lead (Pb)	ND	ND	0.027	ND	0.006	0.022
	Mercury (Hg)	ND	ND	ND	ND	0.014	0.047
	Nickel (Ni)	<LOQ	<LOQ	0.274	<LOQ	0.028	0.095
	Stannum Tin (Sn)	ND	ND	ND	ND	0.014	0.047
Cooked patients’ meals							
Rice	Arsenic (As)	ND	<LOQ	<LOQ	ND	0.019	0.066
	Cadmium (Cd)	0.17	ND	<LOQ	ND	0.017	0.059
	Lead (Pb)	ND	<LOQ	<LOQ	ND	0.006	0.022
	Mercury (Hg)	ND	ND	ND	ND	0.014	0.047
	Nickel (Ni)	0.14	1.18	0.32	0.11	0.028	0.095
	Stannum Tin (Sn)	ND	ND	ND	ND	0.014	0.047
Vegetables	Arsenic (As)	ND	ND	<LOQ	ND	0.019	0.066
	Cadmium (Cd)	ND	ND	ND	ND	0.017	0.059
	Lead (Pb)	ND	ND	ND	ND	0.006	0.022
	Mercury (Hg)	ND	ND	ND	ND	0.014	0.047
	Nickel (Ni)	0.35	0.12	0.22	0.12	0.028	0.095
	Stannum Tin (Sn)	0.064	0.068	ND	<LOQ	0.014	0.047
Soup	Arsenic (As)	ND	ND	ND	ND	0.019	0.066
	Cadmium (Cd)	ND	ND	ND	ND	0.017	0.059
	Lead (Pb)	ND	ND	ND	ND	0.006	0.022
	Mercury (Hg)	ND	ND	ND	ND	0.014	0.047
	Nickel (Ni)	<LOQ	<LOQ	0.2	0.29	0.028	0.095
	Stannum Tin (Sn)	0.13	ND	<LOQ	<LOQ	0.014	0.047
Salad	Arsenic (As)	<LOQ	ND	<LOQ	<LOQ	0.019	0.066
	Cadmium (Cd)	<LOQ	<LOQ	<LOQ	<LOQ	0.017	0.059
	Lead (Pb)	0.111	0.029	0.085	0.023	0.006	0.022
	Mercury (Hg)	<LOQ	<LOQ	<LOQ	ND	0.014	0.047
	Nickel (Ni)	0.166	0.139	0.225	0.228	0.028	0.095
	Stannum Tin (Sn)	ND	0.159	ND	ND	0.014	0.047
Fish	Arsenic (As)	2.13	0.98	0.96	2	0.019	0.066
	Cadmium (Cd)	<LOQ	<LOQ	<LOQ	<LOQ	0.017	0.059
	Lead (Pb)	<LOQ	<LOQ	ND	0.03	0.006	0.022
	Mercury (Hg)	<LOQ	< LOQ	0.08	0.08	0.014	0.047
	Nickel (Ni)	<LOQ	1.09	0.13	0.52	0.028	0.095
	Stannum Tin (Sn)	<LOQ	ND	ND	ND	0.014	0.047

The unit of measurement for heavy metals is milligram mg/kg. ND, Not detected; LOD, Limit of Detection; LOQ, Limit of Quantification (the limit that is considered).

**Table 4:** Assessment of pesticide residue levels (mg/kg) in raw materials and cooked patient meals from two hospitals.

Insecticides	Hospital	Samples from raw materials					Samples from cooked patients’ meals			
		Cucumber (BW)	Cucumber (AW)	Wheat flour	Baked goods (BC)	Baked goods (AC)	Rice	Vegetables	Soup	Salad
Biphenyl	A	0.0349	<LOQ	<LOQ	0.034	<LOQ	<LOQ	<LOQ	<LOQ	<LOQ
	B	<LOQ	<LOQ	<LOQ	0.021	<LOQ	<LOQ	<LOQ	<LOQ	<LOQ
Cyfluthrin 1	A	<LOQ	<LOQ	<LOQ	0.021	<LOQ	<LOQ	<LOQ	<LOQ	<LOQ
	B	<LOQ	<LOQ	<LOQ	<LOQ	<LOQ	<LOQ	<LOQ	<LOQ	0.0157
Cyfluthrin 2	A	<LOQ	<LOQ	<LOQ	0.021	<LOQ	<LOQ	<LOQ	<LOQ	<LOQ
	B	<LOQ	<LOQ	<LOQ	<LOQ	<LOQ	<LOQ	<LOQ	<LOQ	0.0213
Cyfluthrin 3	A	<LOQ	<LOQ	<LOQ	0.021	<LOQ	<LOQ	<LOQ	<LOQ	<LOQ
	B	<LOQ	<LOQ	<LOQ	<LOQ	<LOQ	<LOQ	<LOQ	<LOQ	0.0368
Cyfluthrin 4	A	<LOQ	<LOQ	<LOQ	0.021	<LOQ	<LOQ	<LOQ	<LOQ	<LOQ
	B	<LOQ	<LOQ	<LOQ	<LOQ	<LOQ	<LOQ	<LOQ	<LOQ	0.0372
Deltamethrin	A	<LOQ	<LOQ	<LOQ	0.021	<LOQ	<LOQ	<LOQ	<LOQ	<LOQ
	B	<LOQ	<LOQ	<LOQ	<LOQ	<LOQ	<LOQ	<LOQ	<LOQ	0.0427
Tebuconazole	A	<LOQ	<LOQ	<LOQ	0.021	<LOQ	<LOQ	<LOQ	<LOQ	<LOQ
	B	<LOQ	<LOQ	<LOQ	<LOQ	<LOQ	<LOQ	<LOQ	<LOQ	0.262
Metalaxyl	A	<LOQ	0.0372	<LOQ	<LOQ	<LOQ	<LOQ	<LOQ	<LOQ	0.0172
	B	0.0125	< LOQ	<LOQ	<LOQ	<LOQ	<LOQ	<LOQ	<LOQ	<LOQ
Metalaxyl	A	0.0351	0.054	< LOQ	<LOQ	<LOQ	<LOQ	<LOQ	<LOQ	<LOQ
	B	0.0403	0.0214	< LOQ	<LOQ	<LOQ	<LOQ	<LOQ	<LOQ	<LOQ
Myclobutanil	A	<LOQ	<LOQ	<LOQ	<LOQ	<LOQ	<LOQ	<LOQ	<LOQ	0.017
	B	<LOQ	<LOQ	<LOQ	0.021	<LOQ	<LOQ	<LOQ	<LOQ	<LOQ
Metazachlor	A	0.0172	<LOQ	<LOQ	<LOQ	<LOQ	<LOQ	<LOQ	<LOQ	<LOQ
	B	<LOQ	<LOQ	<LOQ	0.021	<LOQ	<LOQ	<LOQ	<LOQ	<LOQ
Pyridaben	A	<LOQ	<LOQ	<LOQ	<LOQ	<LOQ	<LOQ	<LOQ	<LOQ	<LOQ
	B	0.075	<LOQ	<LOQ	<LOQ	<LOQ	<LOQ	<LOQ	<LOQ	<LOQ
Triadimenol	A	<LOQ	<LOQ	<LOQ	<LOQ	<LOQ	<LOQ	<LOQ	<LOQ	<LOQ
	B	0.0682	<LOQ	<LOQ	<LOQ	<LOQ	<LOQ	<LOQ	<LOQ	<LOQ

The unit of pesticide residuals measurement is milligram mg/kg. ND, Not detected; BC, Before cutting; AC, After cutting; LOD, Limit of detection = 0.05 mg/kg; LOQ, Limit of quantification (the limit that is taken into account) = 0.01 mg/kg.

The results demonstrate that the wheat flour used in both hospital kitchens adheres to food safety standards regarding aflatoxin contamination. Maintaining food safety is critical, particularly in hospital environments where patients are vulnerable. Aflatoxins can have serious long-term health consequences, especially for individuals with compromised immune systems, which are common in hospital settings (Lkhaasuren et al., 2016; Chi et al., 2020). While the findings are positive, it is essential to maintain a proactive approach. Factors like improper storage or procurement from contaminated sources could lead to future aflatoxin contamination. Continued vigilance and adherence to HACCP protocols, as implemented in the hospitals under study, are essential to ensuring ongoing food safety (Rajarajan et al., 2013).

The present study also investigated the concentrations of various heavy metals (arsenic, cadmium, lead, mercury, nickel, and tin) in fresh raw food materials and cooked patient meals from two different hospitals (Table 3). The aim was to assess the potential exposure of patients to these toxic metals through their hospital meals. The samples of raw materials (vegetables, wheat flour, chicken, meat, fish, and eggs) and cooked meals (rice, vegetables, soup, salad, and fish) were collected from the hospitals to determine the limits of detection (LOD) and quantification (LOQ) for each metal. These findings highlight critical information regarding toxic metals, which could pose significant health risks, particularly for vulnerable hospital patients. While most metals were not detected in the samples, the presence of arsenic, mercury, and lead in some food items warrants concern due to their potential long-term health impacts.

The analysis showed varying levels of heavy metal contamination across food types (raw and cooked). Fresh raw fish samples exhibited the highest levels of arsenic (ranging from 0.46 to 1.61 mg/kg) and mercury (up to 0.07 mg/kg), which are known to be highly toxic. Both metals bioaccumulate in aquatic environments, posing a significant long-term health risk when consumed regularly. Fish is a common component of hospital meals, and these findings indicate a potential health risk, particularly for patients with weakened immune systems or chronic conditions, who may be more susceptible to cumulative exposure (Andayesh et al., 2015). Additionally, eggshells showed detectable nickel levels, and low cadmium and nickel levels were found in the internal contents, suggesting minimal contamination risk compared to other animal products (El-Ansary, 2021).

The levels of heavy metals in cooked meals provide insight into the effects of cooking processes on metal concentration. Cooked rice retained low levels of cadmium and nickel, while vegetables showed minimal contamination. This indicates that cooking may reduce heavy metal concentrations in some food types, but in the case of fish, arsenic and mercury remained consistent from raw to cooked samples, suggesting that cooking does not eliminate these metals (Talab et al., 2014). The presence of lead and nickel in various samples further highlights the need for proper sourcing and handling practices to prevent heavy metal contamination. Even though these levels were generally below regulatory thresholds, their potential cumulative effect from long-term exposure must be carefully managed, especially in vulnerable populations.

The results underscore the importance of ongoing monitoring and controlling heavy metal contamination in food served to hospital patients. Even though many of the detected levels were within established safety limits, arsenic, mercury, and lead in fish and certain cooked food items pose long-term exposure risks, particularly for patients with chronic illnesses or weakened immune systems (Letuka et al., 2023). Hospitals must implement stringent quality control measures, including regular testing for heavy metals in both raw materials and cooked foods, to reduce the risk of toxic exposure. Additionally, ensuring proper sourcing of ingredients, particularly fish and animal products, is essential for minimizing heavy metal contamination risks. Strict adherence to HACCP protocols should be maintained to mitigate these risks effectively and ensure food safety for all patients (Sakina et al., 2023).

The study also investigated the levels of various pesticide residues in food samples collected from two hospitals. Quantitative analysis was conducted to determine the concentrations of 12 different insecticides and fungicides, including Biphenyl, Cyfluthrin (4 isomers), Deltamethrin, Tebuconazole, Metalaxyl, Myclobutanil, Metazachlor, Pyridaben, and Triadimenol (Table 4). The food samples included raw materials (cucumber and wheat flour) and cooked patients’ meals (baked goods, rice, vegetables, soup, and salad). Metalaxyl was detected in cucumber samples from both hospitals for the raw materials, with levels ranging from 0.0214 mg/kg to 0.054 mg/kg. Metazachlor was also found in cucumber samples from Hospital A at 0.0172 mg/kg. No pesticide residues were detected above the LOQ in wheat flour samples, suggesting that this staple ingredient in hospital meals is mainly free from chemical contamination (Inobeme et al., 2020). These findings emphasize the need for strict food safety and hygiene standards in handling and preparing food, emphasizing the urgent need for monitoring and control measures for raw materials.

On the other hand, cooked patients’ meals data presented that Biphenyl was detected in baked goods from both hospitals, with concentrations of 0.034 mg/kg in Hospital A and 0.021 mg/kg in Hospital B. Cyfluthrin (all four isomers) and deltamethrin were also found in baked goods from Hospital A at 0.021 mg/kg each, indicating pesticide residues in processed food. Tebuconazole was detected in salad samples from Hospital B at 0.262 mg/kg, the highest concentration among all analyzed food items. Myclobutanil was present in salad samples from Hospital A at 0.017 mg/kg. These elevated levels of fungicides in fresh salads could be a concern, as they indicate pesticide residues in food items commonly served uncooked. Pesticide residues were detected at deficient levels in different categories, such as rice, vegetables, soup, and salad, either below the LOQ or at insufficient levels, posing a minimal risk (Riyaz et al., 2021).

Detection of pesticide residues in raw materials and cooked hospital meals has significant implications for patient health and safety, particularly in hospitals where individuals are more vulnerable to chemical exposures. While most pesticide levels were within acceptable limits, detecting fungicides such as tebuconazole and myclobutanil in salads and insecticides in baked goods highlights potential concerns for long-term exposure. The highest pesticide residue found was tebuconazole in salad samples from Hospital B (0.262 mg/kg), indicating better control over raw ingredient sourcing and washing processes before serving these foods to patients (Riyaz et al., 2021). Additionally, the findings emphasize the importance of continuous monitoring of both raw materials and cooked meals to ensure that pesticide residues remain within safe limits. Hospitals must implement strict procurement guidelines, particularly for fresh vegetables and grains, to reduce the risk of pesticide contamination in patient meals (Hua and Liu, 2024).

### 3.3 Physical analyses

This study assesses physical hazards in healthcare facilities, such as food preparation, handling, and service (Figure 1). Six critical control points (CCPs) were identified, including contamination of raw materials, foreign objects during freezing, food item contamination, prepared food contamination, patient meal contamination, and issues with serving utensils/containers (Edris et al., 2020). The hazards, severity, likelihood, and overall risk levels were systematically assessed. Comprehensive mitigation strategies were developed, emphasizing preventive maintenance, regular inspections, pest control, equipment sanitation, and robust cleaning protocols. The findings offer a practical framework for hospitals to enhance food safety management systems.

Risk levels and control measures were classified using a risk assessment matrix, and our study classified risks based on severity and likelihood. High-level risks were associated with manual handling and equipment malfunction, while environmental factors, such as poor lighting and ventilation, contributed to moderate risks. To mitigate these hazards, our findings recommended: (1) Preventive maintenance and inspections and regular maintenance of kitchen tools and equipment to prevent contamination from broken machinery. (2) Sanitation protocols and strict cleaning of kitchen spaces, utensils, and storage containers to ensure food safety. (3) Continuous pest control strategies to prevent foreign objects from entering the food supply chain (Xiangmo et al., 2014; Iulietto and Evers, 2024).

Our study highlighted the importance of physical hazard management in hospital food safety. Given the vulnerability of hospital patients, even minor foreign object contamination can lead to severe health consequences. Identifying the six CCPs highlights the necessity of rigorous control measures at each stage, from raw material procurement to patient meal service. Proactive risk management strategies—including regular equipment maintenance, robust cleaning protocols, and staff training—are essential to minimizing physical contamination risks (Teffo and Tabit, 2020; Odonkor and Odonkor, 2020).

Table 5 comprehensively analyzes the temperature profiles of various meal components served to patients at two hospitals. It highlights critical insights into the thermal management of these meals throughout their preparation, transport, and distribution. Both hospitals maintained the initial preparation zone at a stable 0°C, ensuring effective cooling before the meal components were assembled and transported. This highlights reasonable initial control of the critical cooling process, preventing early microbial growth. During in-cart transport, the meals experienced notable heat loss. In Hospital A, rice temperatures dropped from 99°C at the cooking stage to 61°C, while in Hospital B, they fell from 96°C to 35°C. Vegetables and meat showed a similar decline, with vegetables dropping from 85.6°C to 59°C in Hospital A and from 81.5°C to 35°C in Hospital B. These significant drops suggest that thermal insulation during transit may be insufficient, especially in Hospital B. The most considerable temperature drops occurred during meal distribution. In Hospital B, rice temperatures plummeted to 33°C, while soup temperatures fell to 30°C, emphasizing inadequate heat retention.

**Table 5:** Tracking thermal profiles of patients’ meals and meal service in two hospitals.

Samples from patients’ meals	Samples observation	Hospital A		Hospital B	
		Temperature (°C)		Temperature (°C)	
		Initial point	Delivery point	Initial point	Delivery point
Initial preparation zone	During patients’ meal line	0.0	0.0	0.0	0.0
	In the cart	20.0	20.0	55.0	68.0
	During distribution	20.0	20.0	6.0	2.0
Juice	During patients’ meal line	6.0	8.5	9.0	9.0
	In the cart	8.0	9.3	15.0	14.0
	During distribution	10.0	11.0	15.0	15.0
Dairy products	During patients’ meal line	6.0	9.0	9.0	6.0
	In the cart	8.0	9.6	15.0	12.0
	During distribution	11.0	11.6	15.0	15.0
Salad	During patients’ meal line	14.0	16.5	15.0	15.0
	In the cart	11.0	11.0	15.0	14.0
	During distribution	13.0	12.0	15.0	18.0
Rice	Zero Time (Cooked area and cart)	99.0	70.0	96.0	95.0
	During patients’ meal line	68.5	93.0	60.0	70.0
	In the cart	61.0	56.5	35.0	40.0
	During distribution	56.0	48.0	33.0	35.0
Vegetables	Zero Time (Cooked area and cart)	85.6	85.4	81.5	80.0
	During patients’ meal line	83.0	75.0	75.0	70.0
	In the cart	69.0	59.0	45.0	35.0
	During distribution	62.0	49.0	40.0	35.0
Meat	Zero Time (Cooked area and cart)	96.3	95.0	80.0	80.0
	During patients’ meal line	62.0	80.0	50.0	70.0
	In the cart	59.0	61.0	33.0	35.0
	During distribution	51.0	57.0	35.0	32.0
Soup	Zero Time (Cooked area and cart)	85.4	75.0	80.0	80.0
	During patients’ meal line	85.5	89.0	55.0	70.0
	In the cart	75.0	59.0	40.0	30.0
	During distribution	60.0	49.7	40.0	30.0

In contrast, Hospital A demonstrated better thermal control, with rice dropping to 56°C and soup to 60°C (Justesen et al., 2016; Young et al., 2016). The findings indicate that while both hospitals perform well in maintaining low temperatures during the preparation phase, there are substantial weaknesses in thermal retention during transport and distribution, particularly in Hospital B. This creates a risk of food entering the danger zone, where microbial growth could occur, compromising food safety (Nabwiire et al., 2021). The study revealed that food temperature variations during the service chain necessitate strict temperature control to prevent microbial growth (Tang and Li, 2017).

## 4 Conclusion

Hospital food safety remains crucial to patient care, requiring continuous evaluation and improvement. This study comprehensively assessed microbial, chemical, and physical hazards across the food service chains of two hospitals in Al-Ahsa Governorate, Saudi Arabia, focusing on implementing HACCP protocols. The findings underscore hospitals’ need to enhance real-time monitoring of food safety practices, as several critical contamination risks were identified that could compromise patient health.

Identified research gaps and safety challenges:

- Microbial hazards: Despite effective pathogen control for *Salmonella* and *Listeria monocytogenes*, the high total bacterial count (TBC) and coliforms in raw and prepared foods indicate gaps in hygiene management, storage, and cross-contamination prevention. Future studies should explore advanced disinfection technologies and improved monitoring systems to mitigate microbial risks at Critical Control Points (CCPs).
- Chemical contaminants: The detection of heavy metals (arsenic, cadmium, lead) in fish and chicken samples, as well as mycotoxins in wheat flour, highlights an urgent need for stricter supplier screening, regular testing of raw materials, and improved sourcing standards. Additionally, pesticide residues in vegetables and salads call for enhanced washing protocols and agricultural traceability measures.
- Physical hazards: Though less frequent, physical contaminants, such as foreign objects introduced during handling and processing, stress the importance of food handler training, stricter quality control, and mechanized sorting and inspection processes.

Key recommendations for hospital food safety enhancement:

- Strengthening HACCP Implementation: Regular staff training programs on hygiene and CCP monitoring are essential to adhere to HACCP principles consistently. Enhanced supervision in meat cutting, vegetable preparation, and tray dispensing should be prioritized.
- Improving Temperature Control & Storage: Ensuring real-time temperature monitoring in food storage, transport, and serving areas can minimize microbial growth and prevent food spoilage before patient consumption.
- Introducing Advanced Sanitization & Monitoring Technologies: Future research should focus on automated microbial detection, innovative food tracking systems, and novel decontamination methods such as UV light treatment or antimicrobial coatings to improve hospital food safety.
- Enhancing Supply Chain Oversight: More rigorous screening of raw materials for chemical contaminants, better supplier accountability, and standardized testing protocols should be established to prevent hazardous substances from entering the hospital food chain.
- By addressing these challenges, hospitals can significantly reduce the risks of foodborne illnesses, improve patient health outcomes, and establish a safer, more reliable food service system. Continuous audits, strict adherence to HACCP protocols, and collaborations with regulatory bodies will ensure long-term improvements in hospital food safety.

Future studies should explore data-driven food safety management systems and AI-assisted contamination detection to enhance hospital nutrition services’ monitoring efficiency and risk mitigation.

## Data Availability

The original contributions presented in the study are included in the article/Supplementary material, further inquiries can be directed to the corresponding authors.
