# Supplementary material for: Comprehensive risk assessment and control measures in the food service chain of hospitals nutrition department: a case study in Al-Ahsa Governorate, Kingdom of Saudi Arabia
Source: Front Microbiol. 2025 May 21;16:1551446. doi: 10.3389/fmicb.2025.1551446 (PMC12133961; doi:10.3389/fmicb.2025.1551446)
Supplement: Supplementary file 1 [file Table_1.DOCX]

**Table S1.** Microbial load (log x10 cfu/g) analysis of raw meat, vegetable, and bakery materials in hospitals of Al-Hasa Governorate, Saudi Arabia.

| Hospital | Sample Type | | Sub-Sample | | TB | | | TMY | | TC | SS | LM |
| --- | --- | --- | --- | --- | --- | --- | --- | --- | --- | --- | --- | --- |
| raw meat materials | | | | | | | | | | | | |
| HA | M | | S | | 2.92 ± 0.36 BCD | | | 3.47 ± 0.01 A | | 2.22 ± 0.21 ABC | 0.00 | 0.00 |
| HA | M | | W | | 3.03 ± 0.39 ABCD | | | 3.02 ± 0.58 ABC | | 2.74 ± 0.34 AB | 0.00 | 0.00 |
| HA | C | | S | | 3.31 ± 0.18 ABC | | | 1.89 ± 0.48 D | | 2.54 ± 0.44 AB | 0.00 | 0.00 |
| HA | C | | W | | 2.99 ± 1.28 BCD | | | 2.83 ± 0.44 BC | | 2.82 ± 0.21 AB | 0.00 | 0.00 |
| HA | F | | S | | 2.48 ± 0.00 CD | | | 2.77 ± 0.24 BC | | 2.67 ± 0.59 AB | 0.00 | 0.00 |
| HA | F | | W | | 3.91 ± 0.62 A | | | 3.08 ± 0.07 ABC | | 2.70 ± 0.14 AB | 0.00 | 0.00 |
| HA | E | | S | | 0.00 ± 0.00 E | | | 0.00 ± 0.00 F | | 0.00 ± 0.00 E | 0.00 | 0.00 |
| HA | E | | W | | 0.00 ± 0.00 E | | | 0.00 ± 0.00 F | | 0.00 ± 0.00 E | 0.00 | 0.00 |
| HB | M | | S | | 3.11 ± 0.06 ABCD | | | 3.07 ± 0.06 ABC | | 2.39 ± 0.07 ABC | 0.00 | 0.00 |
| HB | M | | W | | 3.79 ± 0.45 AB | | | 2.90 ± 0.67 ABC | | 2.96 ± 0.60 A | 0.00 | 0.00 |
| HB | C | | S | | 2.25 ± 0.09 D | | | 3.25 ± 0.04 AB | | 2.02 ± 0.02 BC | 0.00 | 0.00 |
| HB | C | | W | | 2.61 ± 1.78 CD | | | 3.25 ± 0.27 AB | | 2.93 ± 0.22 A | 0.00 | 0.00 |
| HB | F | | S | | 2.48 ± 0.00 CD | | | 2.51 ± 0.36 C | | 1.59 ± 0.09 CD | 0.00 | 0.00 |
| HB | F | | W | | 3.04 ± 0.62 ABCD | | | 2.55 ± 0.61 C | | 1.57 ± 1.81 CD | 0.00 | 0.00 |
| HB | E | | S | | 0.61 ± 0.71 E | | | 1.06 ± 0.91 E | | 0.81 ± 0.95 DE | 0.00 | 0.00 |
| HB | E | | W | | 0.00 ± 0.00 E | | | 0.00 ± 0.00 F | | 0.00 ± 0.00 E | 0.00 | 0.00 |
| LSD (*p≤*0.05) | | | | | 0.914 | | | 0.583 | | 0.823 | --- | --- |
| raw vegetable materials | | | | | | | | | | | | |
| HA | | CF | | BC | | 2.45 ± 0.66 A | 2.04 ± 0.24 B | | 3.06 ± 0.29 AB | | 0.00 | 0.00 |
| HA | | CF | | AC | | 3.07 ± 0.37 A | 1.87 ± 0.57 B | | 1.50 ± 1.03 B | | 0.00 | 0.00 |
| HB | | CF | | BC | | 2.86 ± 0.17 A | 3.32 ± 0.34 A | | 3.25 ± 0.00 A | | 0.00 | 0.00 |
| HB | | CF | | AC | | 2.62 ± 0.67 A | 2.30 ± 0.68 B | | 1.55 ± 1.79 B | | 0.00 | 0.00 |
| LSD (*p≤*0.05) | | | | | | 1.609 | 0.752 | | 1.609 | | --- | --- |
| raw bakery materials (Wheat Flour) | | | | | | | | | | | | |
| HA | | B | | WF | | 1.65 ± 0.42 A | 2.38 ± 0.73 A | | 2.75 ± 0.68 A | | 0.00 | 0.00 |
| HB | | B | | WF | | 1.44 ± 0.74 A | 2.31 ± 0.47 A | | 1.83 ± 0.12 B | | 0.00 | 0.00 |
| LSD (*p≤*0.05) | | | | | | 1.047 | 1.063 | | 0.839 | | --- | --- |

Values represented are mean of four replicates ± standard division (SD). Means in the same column with the same letters are non-significant at *p* ≤ 0.05 according to least significant difference (LSD) test.

Abbreviations: HA: Hospital A, HB: Hospital B, TB: Total Bacterial Count, TMY: Total Mold and Yeast, TC: Total Coliform, SS: Staphylococcus spp., LM: Listeria monocytogenes, M: Meat, C: Chicken, F: Fish, E: Fresh Egg, B: Bakery, WF: Wheat flour, W: Whole Sample, S: Surface sample, CF: Cucumber Fruit, BC: Before Cutting, AC: After Cutting, CF: Cucumber Fruit.

**Table S2.** Comparative microbial profiles (log x10 cfu/g) of meal preparation components in hospitals of Al-Hasa Governorate, Saudi Arabia.

| Hospital | | Sample Type | | Sub-Sample | | TB | | TMY | | TC | SS | | LM | |
| --- | --- | --- | --- | --- | --- | --- | --- | --- | --- | --- | --- | --- | --- | --- |
| Meal’s preparation | | | | | | | | | | | | | | |
| HA | | S | | W | | 3.26 ± 1.44 A | | 0.00 | | 0.00 | 0.00 | | 0.00 | |
| HA | | CV | | W | | 0.60 ± 0.51 BC | | 0.00 | | 0.00 | 0.00 | | 0.00 | |
| HA | | FS | | W | | 0.66 ± 0.59 BC | | 0.00 | | 0.00 | 0.00 | | 0.00 | |
| HA | | CR | | W | | 0.63 ± 0.89 BC | | 0.00 | | 0.00 | 0.00 | | 0.00 | |
| HA | | CF | | W | | 0.70 ± 0.50 BC | | 0.00 | | 0.00 | 0.00 | | 0.00 | |
| HB | | S | | W | | 0.06 ± 0.13 C | | 0.00 | | 0.00 | 0.00 | | 0.00 | |
| HB | | CV | | W | | 0.92 ± 0.19 BC | | 0.00 | | 0.00 | 0.00 | | 0.00 | |
| HB | | FS | | W | | 1.21 ± 0.82 B | | 0.00 | | 0.00 | 0.00 | | 0.00 | |
| HB | | CR | | W | | 1.24 ± 0.48 B | | 0.00 | | 0.00 | 0.00 | | 0.00 | |
| HB | | CF | | W | | 0.40 ± 0.52 BC | | 0.00 | | 0.00 | 0.00 | | 0.00 | |
| LSD (*p≤*0.05) | | | | | | 0.922 | | --- | | --- | --- | | --- | |
| bakery preparation | | | | | | | | | | | | | | |
| HA | B | | BC | | 0.00 ± 0.00 B | | 0.00 ± 0.00 B | | 1.20 ± 0.25 B | | | 0.00 | | 0.00 |
| HA | B | | aC | | 0.00 ± 0.00 B | | 0.00 ± 0.00 B | | 2.03 ± 0.74 AB | | | 0.00 | | 0.00 |
| HB | B | | BC | | 2.37 ± 0.75 A | | 1.40 ± 0.27 AB | | 2.04 ± 0.52 AB | | | 0.00 | | 0.00 |
| HB | B | | aC | | 2.60 ± 0.60 A | | 1.60 ± 0.54 A | | 2.40 ± 0.10 A | | | 0.00 | | 0.00 |
| LSD (*p≤*0.05) | | | | | 1.345 | | 0.968 | | 1.160 | | | --- | | --- |

Values represented are mean of four replicates ± standard division (SD). Means in the same column with the same letters are non-significant at *p* ≤ 0.05 according to least significant difference (LSD) test.

Abbreviations: HA: Hospital A, HB: Hospital B, TB: Total Bacterial Count, TMY: Total Mold and Yeast, TC: Total Coliform, SS: Staphylococcus spp., LM: Listeria monocytogenes, S: Soap, CV: Cooked vegetables, FS: Fresh salad, CR: Cooked Rice, CF: Cooked Fish, W: Whole sample, B: Bakery, BC: Before Cutting, AC: After Cutting.

**Table S3.** Comparative microbial loads (log x10 cfu/g) in critical control points across hospitals in Al-Hasa Governorate, Saudi Arabia.

| Hospital | Sample Type | | Sub-Sample | TB | TMY | TC | SS | LM |
| --- | --- | --- | --- | --- | --- | --- | --- | --- |
| CCP1 | | | | | | | | |
| HA | RWH | | S | 1.96 ± 1.00 AB | 0.95 ± 0.05 B | 0.00 ± 0.00 C | 0.00 | 0.00 |
| HA | RCU | | S | 1.24 ± 1.00 B | 2.19 ± 0.29 A | 2.24 ± 0.24 A | 0.00 | 0.00 |
| HB | RWH | | S | 0.00 ± 1.00 C | 0.00 ± 0.00 C | 0.00 ± 0.00 C | 0.00 | 0.00 |
| HB | RCU | | S | 3.07 ± 1.00 A | 2.48 ± 0.00 A | 2.89 ± 0.03 B | 0.00 | 0.00 |
| LSD (*p≤*0.05) | | | | 1.194 | 0.230 | 0.306 | --- | --- |
| CCP2 | | | | | | | | |
| HA | CFFR | | S | 0.67 ± 0.29 B | 1.24 ± 0.24 B | 0.00 ± 0.00 B | 0.00 | 0.00 |
| HA | FCR | | S | 2.48 ± 0.00 A | 2.98 ± 0.50 A | 2.66 ± 0.52 A | 0.00 | 0.00 |
| HB | CFFR | | S | 0.00 ± 0.00 C | 0.00 ± 0.00 C | 0.00 ± 0.00 B | 0.00 | 0.00 |
| HB | FCR | | S | 2.48 ± 0.00 A | 2.48 ± 0.00 A | 2.48 ± 0.00 A | 0.00 | 0.00 |
| LSD (*p≤*0.05) | | | | 0.288 | 0.621 | 0.517 | --- | --- |
| CCP3 | | | | | | | | |
| HA | BUH | | SB | 2.14 ± 0.18 E | 2.05 ± 0.55 E | 1.97 ± 0.47 DE | 0.00 | 0.00 |
| HA | BUH | | SA | 2.45 ± 0.17 DE | 2.45 ± 0.49 DE | 2.23 ± 0.42 CD | 0.00 | 0.00 |
| HA | BUM | | SB | 2.73 ± 0.08 CD | 2.55 ± 0.55 CD | 1.89 ± 0.29 DE | 0.00 | 0.00 |
| HA | BUM | | SA | 3.09 ± 0.39 AB | 2.66 ± 0.66 CD | 2.32 ± 0.62 CD | 0.00 | 0.00 |
| HA | BUC | | SB | 2.86 ± 0.22 ABC | 2.45 ± 0.00 DE | 2.12 ± 0.82 CDE | 0.00 | 0.00 |
| HA | BUC | | SA | 2.84 ± 0.37 BC | 3.48 ± 0.00 A | 2.66 ± 0.18 ABC | 0.00 | 0.00 |
| HA | BUF | | SB | 1.00 ± 0.00 G | 0.00 ± 0.00 F | 1.00 ± 0.00 G | 0.00 | 0.00 |
| HA | BUF | | SA | 3.15 ± 0.16 AB | 2.98 ± 0.50 BC | 2.35 ± 0.35 BCD | 0.00 | 0.00 |
| HB | BUH | | SB | 2.466 ± 0.05 BC | 0.00 ± 0.00 F | 0.00 ± 0.00 H | 0.00 | 0.00 |
| HB | BUH | | SA | 2.84 ± 0.01 DE | 0.00 ± 0.00 F | 0.00 ± 0.00 H | 0.00 | 0.00 |
| HB | BUM | | SB | 1.45 ± 0.45 F | 2.62 ± 0.13 CD | 1.15 ± 0.15 FG | 0.00 | 0.00 |
| HB | BUM | | SA | 2.95 ± 0.05 ABC | 3.27 ± 0.00 AB | 1.65 ± 0.05 EF | 0.00 | 0.00 |
| HB | BUC | | SB | 0.00 ± 0.00 H | 2.00 ± 0.04 E | 0.00 ± 0.00 H | 0.00 | 0.00 |
| HB | BUC | | SA | 3.19 ± 0.04 A | 2.98 ± 0.50 BC | 3.18 ± 0.07 A | 0.00 | 0.00 |
| HB | BUF | | SB | 0.00 ± 0.00 H | 0.00 ± 0.00 F | 0.00 ± 0.00 H | 0.00 | 0.00 |
| HB | BUF | | SA | 2.48 ± 0.00 DE | 3.23 ± 0.14 AB | 2.88 ± 0.07 AB | 0.00 | 0.00 |
| LSD (*p≤*0.05) | | | | 0.342 | 0.457 | 0.560 | --- | --- |
| CCP4 | | | | | | | | |
| HA | | BAH | SB | 1.80 ± 0.20 B | 1.94 ± 0.34 AB | 0.00 ± 0.00 | 0.00 | 0.00 |
| HA | | BAH | SA | 2.03 ± 0.29 A | 2.22 ± 0.24 A | 0.00 ± 0.00 | 0.00 | 0.00 |
| HA | | BAC | SB | 0.00 ± 0.00 D | 1.87 ± 0.37 AB | 0.00 ± 0.00 | 0.00 | 0.00 |
| HA | | BAC | SA | 1.00 ± 0.00 C | 2.12 ± 0.64 A | 1.00 ± 0.00 | 0.00 | 0.00 |
| HB | | BAH | SB | 0.00 ± 0.00 D | 1.81 ± 0.31 B | 0.00 ± 0.00 | 0.00 | 0.00 |
| HB | | BAH | SA | 0.00 ± 0.00 D | 1.87 ± 0.43 AB | 0.00 ± 0.00 | 0.00 | 0.00 |
| HB | | BAC | SB | 0.00 ± 0.00 D | 0.00 ± 0.00 C | 0.00 ± 0.00 | 0.00 | 0.00 |
| HB | | BAC | SA | 0.00 ± 0.00 D | 0.00 ± 0.00 C | 0.00 ± 0.00 | 0.00 | 0.00 |
| LSD (*p≤*0.05) | | | | 0.202 | 0.392 | --- | --- | --- |
| CCP5 | | | | | | | | |
| HA | | VHW | SB | 2.23 ± 0.73 C | 1.94 ± 0.46 BC | 0.00 ± 0.00 D | 0.00 | 0.00 |
| HA | | VHW | SA | 2.64 ± 0.63 BC | 2.33 ± 0.37 B | 0.00 ± 0.00 D | 0.00 | 0.00 |
| HA | | VCU | SB | 1.30 ± 0.30 D | 1.55 ± 0.35 C | 0.00 ± 0.00 D | 0.00 | 0.00 |
| HA | | VCU | SA | 2.53 ± 0.53 BC | 2.96 ± 0.46 A | 2.63 ± 0.43 B | 0.00 | 0.00 |
| HB | | VHW | SB | 3.00 ± 0.01 AB | 0.00 ± 0.00 D | 0.00 ± 0.00 D | 0.00 | 0.00 |
| HB | | VHW | SA | 3.43 ± 0.14 A | 0.00 ± 0.00 D | 0.00 ± 0.00 D | 0.00 | 0.00 |
| HB | | VCU | SB | 1.54 ± 0.06 D | 1.97 ± 0.07 B | 1.00 ± 0.00 C | 0.00 | 0.00 |
| HB | | VCU | SA | 3.26 ± 0.18 A | 3.09 ± 0.02 A | 3.26 ± 0.18 A | 0.00 | 0.00 |
| LSD (*p≤*0.05) | | | | 0.574 | 0.406 | 0.275 | --- | --- |
|  |  | |  | CCP6 |  |  |  |  |
| HA | BCH | | S | 2.50 ± 0.30 A | 1.15 ± 0.15 B | 0.00 ± 0.00 B | 0.00 | 0.00 |
| HA | PFC | | S | 1.73 ± 0.23 B | 1.73 ± 0.23 A | 0.00 ± 0.00 B | 0.00 | 0.00 |
| HA | PHD | | S | 1.39 ± 0.09 C | 1.72 ± 0.42 A | 1.64 ± 0.14 A | 0.00 | 0.00 |
| HA | PPP | | S | 0.00 ± 0.00 D | 0.00 ± 0.00 D | 0.00 ± 0.00 B | 0.00 | 0.00 |
| HA | PTS | | S | 0.00 ± 0.00 D | 0.00 ± 0.00 D | 0.00 ± 0.00 B | 0.00 | 0.00 |
| HA | PDS | | S | 0.00 ± 0.00 D | 0.85 ± 0.15 C | 0.00 ± 0.00 B | 0.00 | 0.00 |
| HB | BCH | | S | 0.00 ± 0.00 D | 0.00 ± 0.00 B | 0.00 ± 0.00 B | 0.00 | 0.00 |
| HB | PFC | | S | 0.00 ± 0.00 D | 0.00 ± 0.00 B | 0.00 ± 0.00 B | 0.00 | 0.00 |
| HB | PHD | | S | 0.00 ± 0.00 D | 0.00 ± 0.00 B | 0.00 ± 0.00 B | 0.00 | 0.00 |
| HB | PPP | | S | 0.00 ± 0.00 D | 0.00 ± 0.00 B | 0.00 ± 0.00 B | 0.00 | 0.00 |
| HB | PTS | | S | 0.00 ± 0.00 D | 0.00 ± 0.00 B | 0.00 ± 0.00 B | 0.00 | 0.00 |
| HB | PDS | | S | 0.00 ± 0.00 D | 0.00 ± 0.00 B | 0.00 ± 0.00 B | 0.00 | 0.00 |
| LSD (*p≤*0.05) | | | | 0.188 | 0.228 | 0.068 | --- | --- |

Values represented are mean of four replicates ± standard division (SD). Means in the same column with the same letters are not significant at *p* ≤ 0.05 according to least significant difference (LSD) test.

Abbreviations: HA: Hospital A, HB: Hospital B, TB: Total Bacterial Count, TMY: Total Mold and Yeast, TC: Total Coliform, SS: Staphylococcus spp., LM: Listeria monocytogenes, RWH: Receiving worker's hand, RCU: Containers before unloading from receipt, S: Swap, CFFR: Container from the freezer room, FCR: From the cold room, BUH: Butchers' hands, BUM: Meat cutting board, BUM: Meat cutting board, BUC: Chicken cutting board, BUC: Chicken cutting board, BUF: Fish cutting board, BUF: Fish cutting board, SB: Swap Before use, SA: Swap After use, BAH: Bakers hands, BAC Baking cutting boards, BUH: Butchers' hands, BUM: Meat cutting board, BUM: Meat cutting board, BUC: Chicken cutting board, BUC: Chicken cutting board, BUF: Fish cutting board, BUF: Fish cutting board, S: Swap, CCP1: Critical Control Point of Receiving area, CCP2: Freezer and cooling rooms, CCP3: Butchery area, CCP4: Bakery preparation area, CCP5: Vegetable and salad preparation area, CCP6: Patient Meal Dispensing area.
